# Caecocentral Scotoma: A Rare Presentation of Optic Perineuritis

**DOI:** 10.7759/cureus.6101

**Published:** 2019-11-08

**Authors:** Shahidatul-Adha Mohamad, Embong Zunaina, Wan-Hazabbah Wan Hitam

**Affiliations:** 1 Ophthalmology, School of Medical Sciences, Universiti Sains Malaysia, Kota Bharu, MYS

**Keywords:** optic perineuritis, optic neuritis, caecocentral scotoma, retrobulbar optic neuritis, doughnut sign, tram-track sign

## Abstract

Optic perineuritis (OPN) is a subtype of optic neuritis (ON) in which the inflammatory process involves meningeal sheath surrounding the optic nerve. Clinically, OPN simulates ON. However, in contrast to ON, patient with OPN shows sparing of central vision, improves dramatically with high-dose corticosteroid, are more likely to experience recurrence after stopping treatment. We report a rare case of caecocentral scotoma observed in a female with typical ON symptoms. Her magnetic resonance imaging showed features in line with OPN. She was treated with intravenous methylprednisolone 1 g/day for five days followed by slow tapering dose of oral prednisolone for one month. Her vision improved dramatically with a resolution of visual field defect. No relapses seen within two years of follow-up.

## Introduction

Optic perineuritis (OPN) is a rare form of orbital inflammatory disease. The characteristic inflammation of the optic nerve sheath distinguishes it from optic neuritis (ON), which is frequently associated with multiple sclerosis and neuromyelitis optica (NMO), where the optic nerve itself is inflamed [1,2]. Among patients with confirmed OPN, typically reported visual field defects are paracentral scotoma and arcuate defect, with sparing of central vision [1,3]. Here, we report the unusual presentation of OPN manifested with caecocentral scotoma. Diagnosis of OPN is confirmed by the evidence of positive "doughnut sign" and "tram-track sign" on magnetic resonance imaging (MRI).

## Case presentation

A 42-year-old Malay lady first attended our eye clinic in 2011, following a five-day history of right eye blurring of vision, slight pain on eye movement and dyschromatopsia. Right relative afferent pupillary defect (RAPD) was present. The best corrected visual acuity was 6/60 on the affected eye, 6/6 on the left. No optic disc swelling was observed. Humphrey visual field test showed central scotoma, and MRI of the orbit, brain and spine was otherwise normal. A diagnosis of right retrobulbar ON was established, and corticosteroid treatment was commenced with intravenous melthylprednisolone 250 mg qid for three days followed by oral prednisolone 1 mg/kg/day for 11 days. Post-treatment one month, the visual acuity improved to 6/6, with a complete resolution of central scotoma.

The patient presented again in June 2016 with recurrent symptoms affecting the other eye. The patient complained that they had blurring vision and dyschromatopsia in the left eye, which progressively worsened over a two-week duration. It was associated with mild retro-orbital dull-aching pain on eye movement. There was no other significant ocular, systemic or neurological complaint. There was no suggestive history of infective or connective tissue problems.

**Figure 1:**
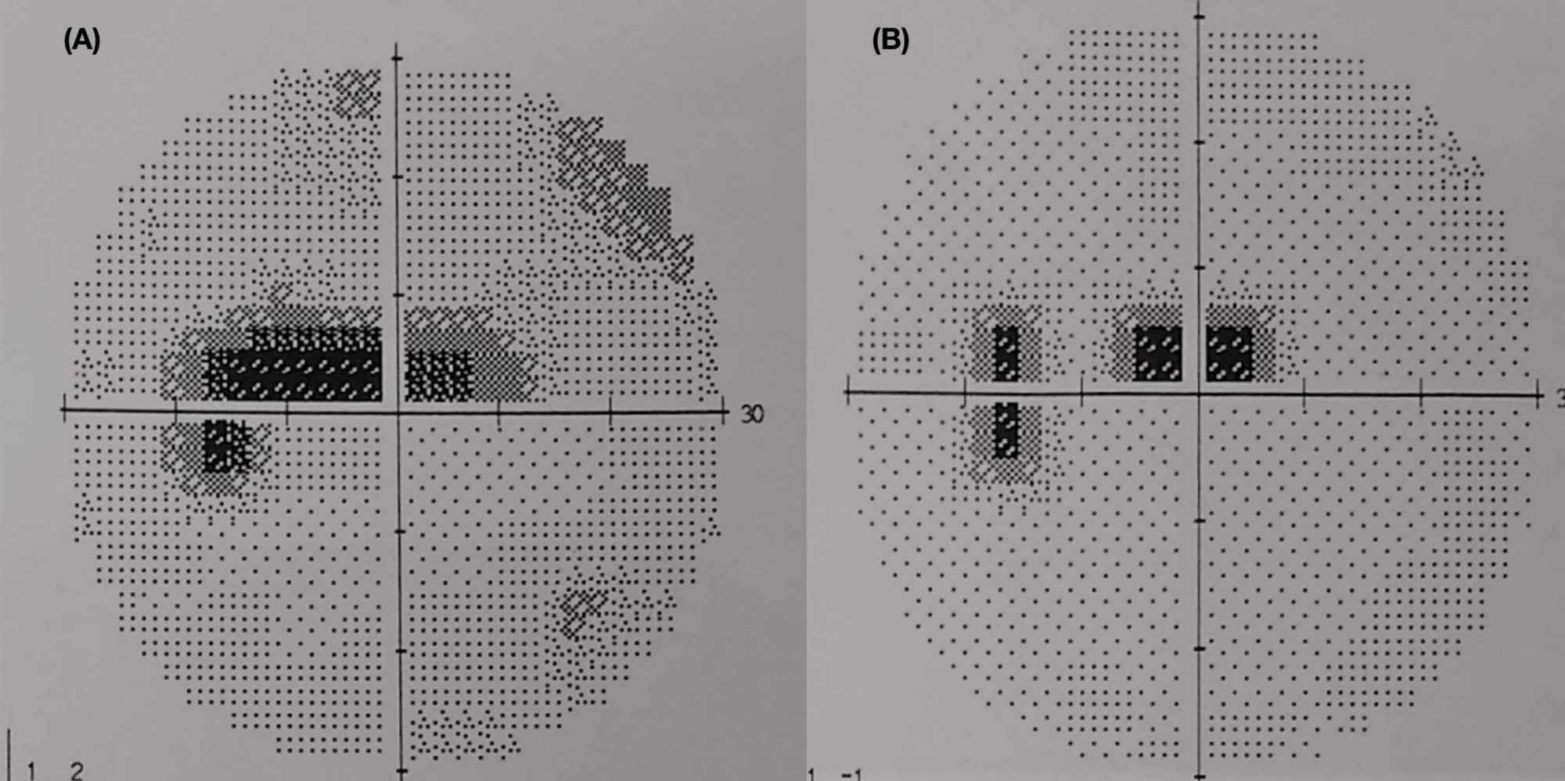
Humphrey visual field of the left eye shows: (A) a caecocentral defect (before treatment), and (B) improving central scotoma (after 3 days of treatment)

The best corrected visual acuity was 6/12 in the left eye and 6/6 in the right. The assessment of the left eye revealed positive RAPD, reduced colour vision and the presence of caecocentral visual field defect (Figure 1). Anterior segment examination was unremarkable, with normal intraocular pressure. Funduscopy revealed hyperaemia of the left optic disc. There was temporal pallor of the right optic disc due to previous ON. The retina and macula were otherwise normal on both eyes.

Laboratory tests returned normal results for blood count, erythrocyte sedimentation rate, blood glucose, renal profile, liver function tests, C-reactive protein, antinuclear antibodies, complements and rheumatoid factor. Additional investigations to exclude other causes of ON such as tuberculosis (Mantoux test, chest x-ray), syphilis (electrochemiluminescence immunoassay), venereal disease research laboratory test, sarcoidosis (serum and urine calcium, serum angiotensin converting enzyme) and NMO (anti-aquaporin 4 antibodies) were all negative. No cerebrospinal fluid analysis or detection of oligoclonal antibodies was carried out, as the patient refused these.

**Figure 2:**
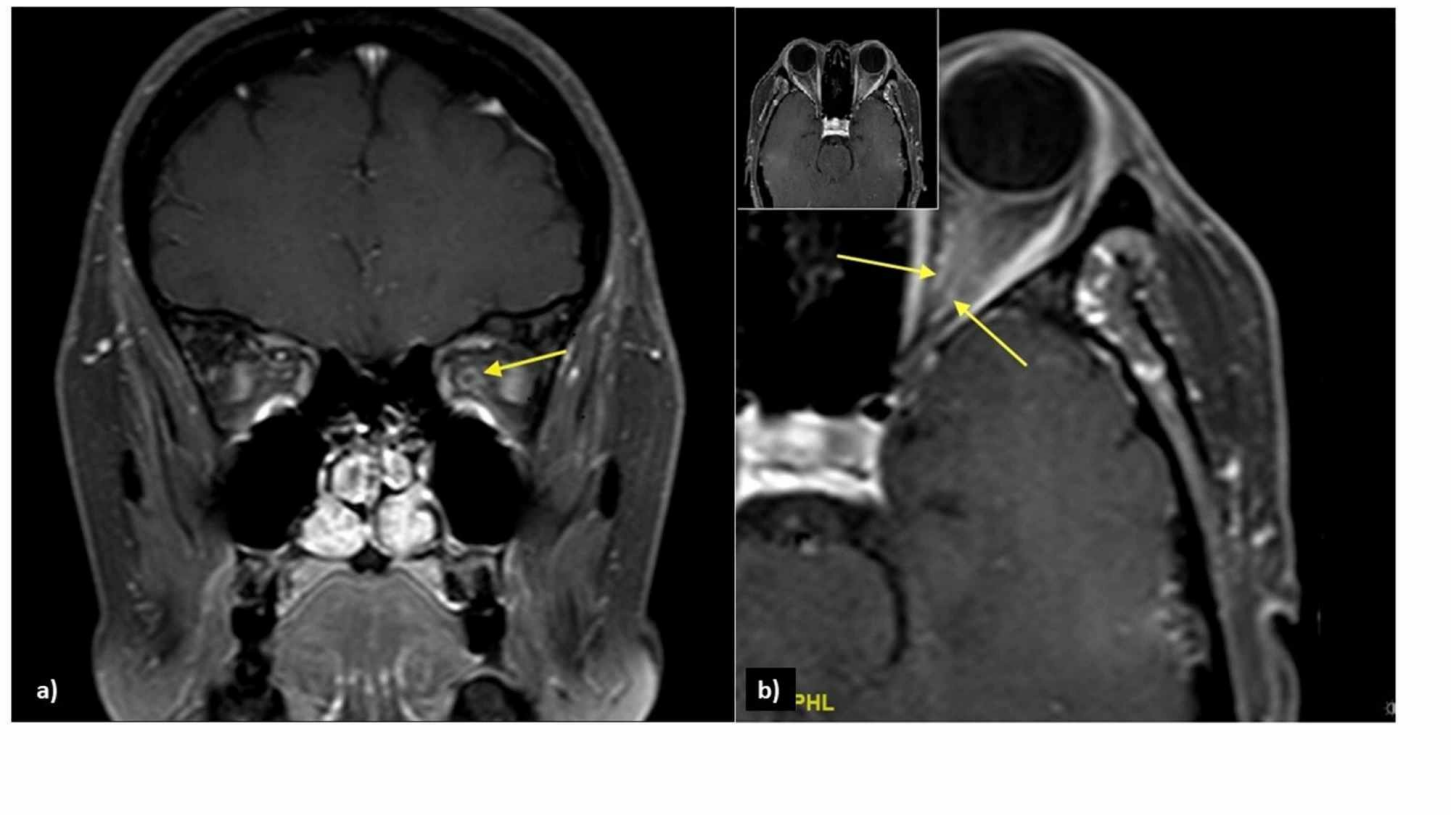
Post-contrast T1-weighted image with fat suppression shows: a) doughnut sign on coronal view (arrow) and b) tram-track sign on axial view (arrows)

An urgent MRI of orbit revealed an enlarged left intra-orbital nerve (0.45 cm) as compared to the right (0.28 cm). The post-gadolinium contrast MRI showed streakiness and enhancement of periorbital fat surrounding the left optic nerve, with tram-track sign on saggital view, and doughnut sign on coronal sequence, consistent with the left OPN (Figure 2). No abnormalities were detected on MRI of the brain and paranasal sinuses.

Based on the positive MRI findings with negative findings on all other investigations, a confirmed diagnosis of left idiopathic OPN was established. The patient was treated with high-dose intravenous methylprednisolone 1 g/day for five days, followed by 1 mg/kg/day of oral prednisolone with tapering dose over one month. The positive response to treatment was observed in the first three days of treatment whereby less central scotoma reported (Figure 1). After four weeks on corticosteroid therapy, the vision of affected eye improved to 6/6, with normal optic nerve function including colour vision and visual field test. 

The optic nerve function was closely monitored during follow-up visits. At two-year follow-up, the patient remains relapse free with good visual function.

## Discussion

Clinically, OPN can be mistaken for ON. The triad of classical optic neuropathy includes vision loss, dyschromatopsia and visual field defect that are compelling in both clinical entities. Ancillary investigations to support the diagnosis and to rule out other possible causes are mandatory as the management and prognosis are different. In the present case, our patient exhibited subacute symptoms that are typical for OPN, but with an uncommon field defect. Her clinical symptoms alarmed us for possibilities of recurrent ON. Meanwhile, her caecocentral field defect is almost similar to hereditary and toxic-nutritional optic neuropathies, which are characterized by selective maculo-papillar bundle involvement except for the unilaterality. To the best of our knowledge, this is the first reported case of OPN with associated atypical caecocentral scotoma.

OPN is an uncommon spectrum of orbital inflammatory pseudotumour that was first described by Edmunds and Lawford in 1883 [1,3]. Extensive literature reviews indicate that OPN has a slower onset than ON, with characteristic paracentral and arcuate defects sparing the central field [1,4,5]. In a review of 15 eyes with OPN, 10 were found to have spared central vision [4]. However, in a recent study of six OPN patients, one was found to have a small isolated central scotoma [5].

MRI (ideally, a fat-suppressed, post-gadolinium contrast) provides absolute detail of classic perineural enhancement as well as subtle enhancement of extraocular muscles and/or sclera and streakiness of orbital fat [4,6,7]. A contrast computed tomography (CT) can also be used to detect the tram-track sign and doughnut sign, but the spatial resolution is insufficient to define perineural from intra-neural enhancement as seen in demyelinating ON [4].

Clinical judgement becomes crucial when CT and MRI are unfeasible, especially in cases of severe vision loss. A definitive treatment with high-dose intravenous corticosteroid is the gold standard in addressing both ON and OPN [7]. However, patients with OPN require a subsequent longer oral corticosteroid therapy with slow tapering of the dose to prevent relapse and recurrence [1,4,8]. The exact duration remains unclear and poorly studied because of the rarity of this disease. Whilst for ON, a widely used regime is based on Optic Neuritis Treatment Trial: intravenous methylprednisolone 1 g/day for three days, followed by 11 days of oral prednisolone 1 mg/kg/day. This accelerates the visual recovery, but it has no impact on the final visual outcome and recurrence rate.

Some studies have suggested that failure to treat or delay in administering high-dose intravenous corticosteroid to OPN patients will result in a poor visual outcome [4]. However, in an observation involving three Japanese patients with OPN, two resolved with good visual outcome despite a six-month delay between first onset of symptoms and initiation of steroid therapy [3]. As optic atrophy had not yet set in, it was concluded that both high-dose corticosteroid therapy and pulse therapy are equally effective when treating new cases of idiopathic OPN or following previously failed moderate-dose corticosteroid treatment.

While most OPN are idiopathic in nature, as in the present case, a thorough evaluation of patients with OPN is nevertheless important in determining a specific secondary etiology such as tuberculosis, syphilis, Wegener granulomatosis, giant cell arteritis, sarcoidosis or systemic lupus erythematosus [1,4,5,9,10]. Specific treatment of such causes is necessary beyond corticosteroid therapy.

## Conclusions

OPN is rare and frequently mistaken for ON. A careful evaluation is therefore essential when dealing with unilateral optic neuropathy with central and caecocentral scotomas. Recognition of definite disease entity ensures a proper management and provides a good visual prognosis.
